# Characterization of the complete chloroplast genome of *Carex myosuroides* Villars, 1779 (Cyperaceae)

**DOI:** 10.1080/23802359.2022.2053368

**Published:** 2022-03-25

**Authors:** Hong-Yan Chen, Xiao-Fei Xia, Zhe Pan, Yu Ning

**Affiliations:** a Beijing Botanical Garden, Beijing, China; b Department of Specimens, Beijing Museum of Nature History, Beijing, China; c Sichuan Academy of Environmental Policy and Planning, Chengdu, China; d Institute of Wetland Research, Chinese Academy of Forestry Research, Beijing, China

**Keywords:** *Carex myosuroides*, Cyperaceae, complete chloroplast genome, alpine

## Abstract

*Carex myosuroides* Villars, 1779 is a typical alpine sedge with both ecological and agricultural value. The work reported here is the first complete chloroplast genome of this species. The chloroplast genome, with a total size of 185,609 bp, consists of two inverted repeats (IRs, 38,374 bp) separated by a large single-copy (LSC, 99,911 bp) region, and a small single-copy (SSC, 8950 bp) region. The overall genome GC content is 34.12%. The genome contains 125 genes, consisting of 82 protein-coding genes, 35 tRNA genes, and eight rRNA genes. Phylogenetic analysis supports the taxonomic treatment of incorporating genus *Kobresia* to a broader circumscription of *Carex*. Our work could be helpful to future research on Cyperaceae.

*Carex myosuroides* Villars, 1779, formerly known as *Kobresia myosuroides* (Villars) Foiri, 1896 (Zhang and Noltie 2010), is a typical sedge in alpine environment. It is the only known sedge to harbor associations with ectomycorrhizal fungi, establishing an effective way of transferring amino acid-nitrogen from the soil solution (Lipson et al. 1999). Thus, it usually appears as the main species in high-altitude vegetation, especially in highland meadow. It is reported that this species also contributes a notable part to the feeding of livestock in Qinghai-Tibet Plateau (Miehe et al. 2019). As alpine ecosystems commonly are considered to be more prone to challenges brought up by climate change, the integrity and vigor of alpine dominant species is of vital importance (Li et al. 2020). However, molecular information to date is limited for alpine sedges. Our work here presents the first annotated full-length assembly of the chloroplast of *C. myosuroides*, which could be helpful to future research on Cyperaceae.

The samples were collected at Dongling mountain, Beijing, China (GPS: E115°30′11″, N 40°3′7″). Fresh leaves were collected and wrapped in drikold, then transferred to be stored at −80 °C lab environment. The relevant specimen was deposited at the herbarium of Beijing Museum of Nature History (http://www.bmnh.org.cn/en/) under the voucher number BJM0271944 (contact xiaxiaofei@bmnh.org.cn). The total genomic DNA was extracted using DP305-3 plant genomic DNA Kit (Tiangen, Beijing, China) according to the standard protocol. The sequencing platform was Illumina NovaSeq 6000. Totally, 19,595,642 high-quality clean reads (PE 150) were generated. Aligning, assembly and annotation were conducted by bowtie2 (v2.2.4) (Langmead and Salzberg 2012), SPAdes (v3.10.1) (Bankevich et al. 2012), SSPACE (v2.0) (Boetzer et al. 2011), and Gapfiller (v2.1.1) (Nadalin et al. 2012). The assembled and annotated chloroplast genome sequence has been submitted to GenBank under the accession number MZ962720.1.

The plastome of *C. myosuroides* is a circular DNA molecule with a length of 185,609 bp. Although this genome size is notably higher than the average genome size of monocots (0.144 M) presented by Mohanta et al. (2020), it is comparable to several published *Carex* chloroplast genomes (∼0.187 M), indicating further investigation is needed for a likely unique evolution trajectory of the sedge group. The full plastome consists of a large single-copy (LSC) region of 99,911 bp, small single-copy (SSC) region of 8950 bp and two inverted repeats (IRs) of 38,374 bp by each. The overall GC content of *C. myosuroides* chloroplast genome was 34.12%, similar to the average level of 36.82% (Mohanta et al. 2020), with corresponding GC values of the LSC, SSC, and IR regions for 32.01%, 26.96%, and 37.69%, respectively. The chloroplast genome of *C. myosuroides* comprises 125 genes, including 82 protein-coding genes, eight ribosomal RNA (rRNA) genes, and 35 transfer RNA (tRNA) genes.

We constructed a maximum likelihood tree to explore the evolutionary position of *C. myosuroides* (Figure 1). In total, 16 complete chloroplast genomes were used to build the phylogeny. Two species from Fabaceae were used as the outgroup (*Astragalus mongholicus* Bunge, 1868 and *Tibetia liangshanensis* Li, Pei Chun, 1981). The sequences were aligned using the default settings in MAFFT v7 (Katoh et al. 2019). The phylogenetic analyses were conducted using IQTREE v1.6.7 (Nguyen et al. 2015). The fitted model was TVM + F+R3, with branch support tested by SH-aLRT (1000 replicates) and ultrafast bootstrap (1000 replicates). Branches were labeled if their SH-aLRT score >85% and ultrafast bootstrap support >75%. Based on the current sampling extent, our result presents a phylogeny with most internal relationships well supported. This result is also in accordance with the taxonomic treatment of incorporating genus *Kobresia* to a broader circumscription of *Carex* (Global Carex Group 2015). Our work could be helpful to further researches in Cyperaceae.

**Figure 1.**
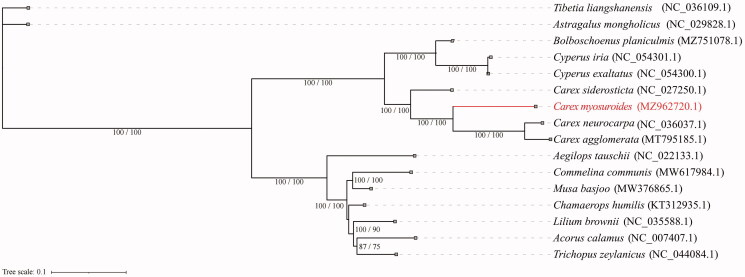
Phylogenetic relationships among 16 complete chloroplast genomes. Branch scores are given as (SH-aLRT value/ultrafast bootstrap value). The analyzed species are shown with the corresponding GenBank accession numbers in parentheses.

## Data Availability

The data that support the findings of this study are openly available in the GenBank database at https://www.ncbi.nlm.nih.gov/, under accession number [MZ962720.1]. The associated BioProject, SRA, and BioSample numbers are PRJNA772905, SRR 16494724, and SAMN 22419664, respectively.
